# Long-term simulation of lead concentrations in agricultural soils in relation to human adverse health effects

**DOI:** 10.1007/s00204-020-02762-x

**Published:** 2020-05-05

**Authors:** Thomas Schupp, Georg Damm, Heidi Foth, Alexius Freyberger, Thomas Gebel, Ursula Gundert-Remy, Jan G. Hengstler, Aswin Mangerich, Falko Partosch, Claudia Röhl, Klaus-Michael Wollin

**Affiliations:** 1 Faculty of Chemical Engineering, Muenster University of Applied Science, Stegerwaldstrasse 39, 48565 Steinfurt, Germany; 2 Department für Hepatobiliäre Chirurgie und Viszerale Transplantation, Universität Leipzig, Liebigstrasse 20, 04103 Leipzig, Germany; 3 Institut für Umwelttoxikologie, Martin Luther Universität Halle, Franzosenweg 1, 06108 Halle (Saale), Germany; 4 Pathology and Clinical Pathology, Bayer Aktiengesellschaft, Aprather Weg 18a, 421113 Wuppertal, Germany; 5 Federal Institute for Occupational Safety and Health, Friedrich-Henkel-Weg 1-25, 44149 Dortmund, Germany; 6 Charité, Institute of Clinical Pharmacology and Toxicology, Universitätsmedizin Berlin, Corporate Member of Freie Universität Berlin, Humboldt-Universität zu Berlin, Berlin Institute of Health, Berlin, Germany; 7 Leibniz-Institut für Arbeitsforschung an der TU Dortmund (IfADo), Ardeystrasse 67, 44139 Dortmund, Germany; 8 Molecular Toxicology Group, Department of Biology, University of Konstanz, Box 628, 78457 Konstanz, Germany; 9 Institut für Arbeitsmedizin, Universitätsmedizin Göttingen, Waldweg 37b, 37073 Göttingen, Germany; 10 Department of Environmental Health Protection, Schleswig-Holstein State Agency for Social Services, 24105 Kiel, Germany; 11 Niedersächsisches Landesgesundheitsamt, 30449 Hannover, Germany

**Keywords:** Lead, Pb, Hunting, Gunshot, Fertilizer, Soil contamination, Food contamination, Consumer risk

## Abstract

Lead (Pb) exposure of consumers and the environment has been reduced over the past decades. Despite all measures taken, immission of Pb onto agricultural soils still occurs, with fertilizer application, lead shot from hunting activities, and Pb from air deposition representing major sources. Little is known about the intermediate and long-term consequences of these emissions. To gain more insight, we established a mathematical model that considers input from fertilizer, ammunition, deposition from air, uptake of Pb by crops, and wash-out to simulate the resulting Pb concentrations in soil over extended periods. In a further step, human oral exposure by crop-based food was simulated and blood concentrations were derived to estimate the margin of exposure to Pb-induced toxic effects. Simulating current farming scenarios, a new equilibrium concentration of Pb in soil would be established after several centuries. Developmental neurotoxicity represents the most critical toxicological effect of Pb for humans. According to our model, a Pb concentration of ~ 5 mg/kg in agricultural soil leads to an intake of approximately 10 µg Pb per person per day by the consumption of agricultural products, the dose corresponding to the tolerable daily intake (TDI). Therefore, 5 mg Pb/kg represents a critical concentration in soil that should not be exceeded. Starting with a soil concentration of 0.1 mg/kg, the current control level for crop fields, our simulation predicts periods of ~ 50 and ~ 175 years for two Pb immission scenarios for mass of Pb per area and year [scenario 1: ~ 400 g Pb/(ha × a); scenario 2: ~ 175 g Pb/(ha × a)], until the critical concentration of ~ 5 mg/kg Pb in soil would be reached. The two scenarios, which differ in their Pb input via fertilizer, represent relatively high but not unrealistic Pb immissions. From these scenarios, we calculated that the annual deposition of Pb onto soil should remain below ~ 100 g/(ha × a) in order not to exceed the critical soil level of 5 mg/kg. We propose as efficient measures to reduce Pb input into agricultural soil to lower the Pb content of compost and to use alternatives to Pb ammunition for hunting.

**Electronic supplementary material:**

The online version of this article (10.1007/s00204-020-02762-x) contains supplementary material, which is available to authorized users.

## Introduction

Lead (Pb) and Pb compounds have been used since ancient times, e.g., in water pipes, roofing, as pigments in paints (carbonate, sulfate, chromate), in ammunition, shielding material against radiation and as weighting material. Pb is highly toxic for humans and still raises major concerns due to its presence in food (WHO/JECFA 2011). Over the last decades, measures were undertaken to reduce Pb exposure. For example, leaded gasoline was phased out during the 1980s in many industrialized countries. As a result, blood Pb concentrations of the general population decreased (Wietlisbach et al. 1995; Pirkle et al. 1994). Other applications of Pb, e.g., in solders for drinking water pipe plumbing, for roofing, in pigments in paints, in toys, and in ceramics for food contact, were increasingly regulated or banned in the European Union (EU). Use in paint has been restricted in the EU (see Regulation [EU] No. 1907/2006, Annex XVII), but painted material containing Pb pigments is still in use and may lead to intoxications (O’Connor et al. 2018). All these regulatory activities have resulted in reduced Pb immissions into the environment and reduced Pb burden in environmental media. From 1990 to 2015, declining background deposition of Pb could be demonstrated by Pb-monitoring in earthworms (LUBW 2006) and in moss species (Schröder and Nickel 2019). Human biomonitoring reference values for Pb in blood of children in Germany declined from 60 µg/L in 1992 to 50 µg/L in 2003, and finally, 36.3 µg/L in 2009 (Umweltbundesamt 1998, 2005,2009). Permitted immission and concentration in soil for different contaminants are regulated on national levels. For example, the German Federal Soil Protection and Contaminated Sites Ordinance (*Bundesbodenschutz-Verordnung*) addresses Pb with control levels (*Prüfwert*) of 400 mg/kg for residential areas, 200 mg/kg for children’s playgrounds, 1200 mg/kg for meadows, and 0.1 mg/kg for crop-fields. If the control level is exceeded, further measures have to be taken to identify and manage potential risks. Precautionary levels named in this ordinance define concentrations which indicate a risk if exceeded; for Pb, these levels are 40, 70 and 100 mg/kg for sand-, silt- and clay-soil, respectively. The annual emission of Pb on soil should not exceed 400 g/ha (BMJV 2017). Despite all measures taken to address and reduce emissions of, and exposure to Pb, the European Food Safety Administration (EFSA) reports oral Pb exposure via food that deserve further attention (EFSA 2010, 2012).

Little is known if current immission of Pb onto farmland is high enough to cause an increase of Pb concentrations over longer periods. To answer this question, we established a mathematical model that allows long-term simulations of Pb concentrations in soil for specific input scenarios. We addressed the question what level of Pb input would lead to concentrations where adverse effects for humans can no longer be excluded.

## Methods

### The model

**Fig. 1:**
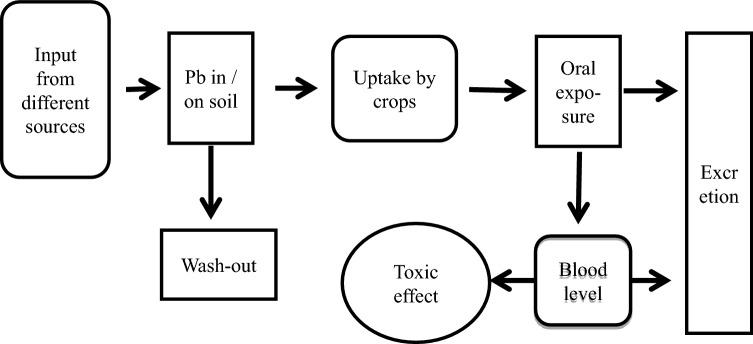
Model for human oral exposure via vegetable products and crops by dispersive deposition of Pb on agricultural soil

Figure 1 depicts schematically the modeling of the scenario to estimate potential future trends for oral Pb exposure via crop intake. In brief, the input into soil was treated as first-order reaction kinetics, and the Pb uptake by plants was calculated by distribution models. An equipartitioning of Pb in the plant tissue was assumed and leads to calculated Pb contents in edible parts of the plants. Daily crop intake data were used to estimate the daily oral Pb exposure from crops. The following stepwise approach was applied:

Step 1: For a hypothetical agricultural field, the initial concentrations of Pb were set to the control levels of the German Soil Protection Act and its subsequent Ordinances, which are 0.1 mg/kg for crop fields (soil scenario 1), or 70 mg/kg, which is the precautionary level for silt soils (soil scenario 2); silt soils are preferred, fertile farmlands. The simulated input of Pb per time and surface area can occur as deposition of rural background levels of Pb in air, via Pb-contaminated fertilizer and by deposition of gunshot.

Step 2: For the assessment of Pb balance in soil, the soil content was calculated as a relation between a static, continuous input and the output by wash-out and plant uptake (plant remediation).

Step 3: The uptake of Pb by plants was calculated depending on soil concentrations for different types of crops. The calculation was based on published data for Pb uptake and crop yield per surface area.

Step 4: Oral Pb exposure via crops was estimated based on the average vegetarian dietary habits for adults, or based on the recommended children food basket for children of 20 kg body weight (b.w.), multiplied with the calculated Pb contents in the different crops from Step 3. It is assumed that cooking does not reduce the Pb load in plant food.

Step 5: The most sensitive effects for Pb toxicity were considered, and associated Pb blood levels were matched against oral exposure as derived in Step 4.

### Lead input

Several possible sources of Pb input into soil were considered. Dust and aerosols may contain Pb due to natural, geological presence and/or due to industrial activities; wet and dry deposition of this material is termed as “immission” in this document. Background deposition of Pb in rural areas of Germany is in a range of 8 – 14 g/(ha × *a*) (Schaap et al. 2018), and the median of 11 g/(ha × *a*) was used for the calculations in this report.

Hunting with gunshot is the second contributor for Pb input into soil modeled in this document. In the EU, 14,000 t/a Pb are dispersed annually as gunshot in hunting areas; another 10,000–20,000 t/a are expected to be deposited on shooting ranges (ECHA 2018b). Gunshot is typically used for game that is hunted on fields and meadows. If the 14,000 annual tons gunshot estimated by ECHA are dispersed over the agricultural surface area of the EU which is 174 million hectare (EC 2017), this would result in an annual load of 80 g/(ha × *a*).

The use of fertilizer is the third identified source of Pb input into soil. For Germany, a detailed analysis of fertilizer use was performed by Knappe et al. (2008); the authors divided conventional farming into four scenarios:

- Use of cattle manure and mineral fertilizer.
- Use of compost and mineral fertilizer.
- Use of sewage sludge and mineral fertilizer.
- Exclusive use of mineral fertilizer.

Data for annual Pb input on soil via fertilizer in dependence on the farming scenario are presented in Table 1.

**Table 1:** Pb input via fertilizer in dependence on farming category according to Knappe et al. (2008)

Farming Scenario	Pb [g/(ha × a)]	
	Median	90-Percentile
A	7.5	9.4
B	313.0	314.6
C	69.5	80.7
D	6.2	7.8

### Lead uptake by crop plants

For calculation of the Pb balance in soil, knowledge of the extent of Pb uptake by plants is required. Extractable Pb in soil is known to correlate with Pb in plant tissue, and a linear relation between Pb in soil and Pb in plants has been reported (Dudka et al. 1996; Attanayake et al. 2014):

1 $${\text{barley grain:}}\;\left[ {{\text{Pb}}} \right]_{{{\text{plant}}}} = 0.0003 \times \left[ {{\text{Pb}}} \right]_{{{\text{soil}}}} .$$

2 $${\text{leaf vegetable:}}\; \left[ {{\text{Pb}}} \right]_{{{\text{plant}}}} = 0.01 \times \left[ {{\text{Pb}}} \right]_{{{\text{soil}}}} .$$

3 $${\text{potato tuber}},{\text{ peeled:}}\;\left[ {{\text{Pb}}} \right]_{{{\text{plant}}}} = 0.003 \times \left[ {{\text{Pb}}} \right]_{{{\text{soil}}}} .$$

4 $${\text{other vegetables:}}\;\left[ {{\text{Pb}}} \right]_{{{\text{plant}}}} = 0.0008 \times \left[ {{\text{Pb}}} \right]_{{{\text{soil}}}} .$$

For Pb removal from soil, only edible parts of plants were taken into account, as the other parts of the plants are assumed as being left on the soil. The removal of Pb from soil by plants is not only solely dependent on the uptake factor of the different plant species, but also a function of the surface density of the plant species, expressed as kg biomass per m^2^ (yield, *Y*), and its share between all other plants on the surface. For our simulations, we assume that the plant species seeded on farmland are selected in such a way that the plant food demand for an adult vegetarian is matched. For example, Table 2 shows daily food intake (DI) for adult vegetarians, as listed by EFSA (2010; see Table 21) for such crops for which plant uptake factors from soil are available. The corresponding required surface of farm or gardening land to supply the daily amount of the respective plant is calculated, and the sum of all modeled plant species covering this land is set to 100% (or factor 1.0). For balancing Pb per m^2^ land, plant uptake is a sink for Pb. As different plant species have different Pb uptake capabilities, the average agricultural soil surface coverage was assumed to be 67.7% for cereals, 3.6% for potatoes, 20.3% for leaf vegetables and 8.4% for other vegetables (UK GOV 2018).

**Table 2:** Median annual Pb input by air deposition, gunshot and fertilizer in dependence on farming category according to Knappe et al. (2008)

Farming Scenario	Pb input (IN)	
	mg/m^2^	mg/kg
A	9.85	0.029
B	40.4	0.119
C	16.1	0.047
D	9.9	0.029

As illustrated in Table 4, an adult vegetarian consumes 0.283 kg cereals per day. As the annual harvest (yield, *Y*) for cereals is 0.8 kg/(m^2^ × *a*), 0.354 m^2^ are required for the daily demand (Table 3). With respect to the area required for the other crops listed in Table 3, these 0.354 m^2^ make up 67.7% of the required farmland to satisfy the daily demand. This results in a surface weighting factor for cereals of P = 0.677.

### Wash-out of lead in soil

Pb in soil may be washed out by irrigating water. The wash-out is the sum of infiltration and run-off, as soluble Pb compounds may leave the soil layer and are no longer available for plant uptake.

Rooney et al. (2007) investigated the correlation of Pb solubility in agricultural soil pore water with the soil pH. The soils were spiked with lead shot. At pH values of 6.9 and 5.7, the concentration of Pb in soil water reached values of approximately 0.5 and 2.0 mg/L, respectively. The field measurements published by Hawkins et al. (1995) were used to estimate the losses of Pb in soil due to wash-out (WO); here, WO is the sum of leaching and wash-off. Leaching and wash-off resulted in an annual loss of approximately 0.2% of the Pb in soil, which is equivalent to a rate constant of 2.0 × 10^–3^∙*a*^−1^.

5 $${\text{WO}} = {2}.0 \times {1}0^{{ - {3}}} \times \left[ {{\text{Pb}}} \right]_{{{\text{soil}}}} \left[ {{\text{mg}}/\left( {{\text{kg}} \times {\text{a}}} \right)} \right].$$

### Lead balance in soil

For the uptake by plants, only those parts which serve as food were modeled since the other parts would most likely end up as fertilizer, i.e., Pb remains in the soil matrix. For removal from farmland, only uptake in edible parts were modeled. The total removal per surface area and time is dependent on the plant type (i), its individual plant uptake factors (UF_i_), its prevalence per surface area (*P*_i_, between 0 and 1) and its yield per time (*Y*_i_). For the balance of Pb in soil per m^2^, the following algorithm was used:

6 $${\text{d}}\left[ {{\text{Pb}}} \right]_{{{\text{soil}}}} /{\text{dt}} = {\text{IN}}{-}\left\{ {{\text{WO}} + \sum_{i} \left( {{\text{UF}} \times P \times Y} \right)_{{\text{i}}} } \right\} \times \left[ {{\text{Pb}}} \right]_{{{\text{soil}}}} .$$

where IN is the input of Pb on soil per area and time. With data presented in Table 3, the last term in Eq. (6) can be calculated as

$$\sum_{i} \left\{ {{\text{UF}} \times P \times Y} \right\}i = {3} \times {1}0^{{ - {4}}} \times 0.{8} \times 0.{677} + {1}0^{{ - {2}}} \times {5}.0 \times 0.0{36} + {3} \times {1}0^{{ - {3}}} \times {3}.0 \times 0.{2}0{3} + {8} \times {1}0^{{ - {4}}} \times {3}.{3} \times 0.{2}0{3} \times 0.0{84} = {4}.0{1} \times {1}0^{{ - {3}}} {\text{a}}^{{ - {1}}} .$$

**Table 3:** Crop yield [*Y*; kg/(m^2^ × a)], Pb uptake factor (UF) and soil surface (m^2^) required to supply plant food for the daily intake for an adult vegetarian (EFSA 2010), and surface weighting factor *P* (*P*_*i*_ = m^2^_*i*_ /∑m^2^_*i*_)

Species	UF	*Y*	m^2^	*P*
Cereals	3.0E-04_soil_^a^	0.8^c^	0.354	0.677
Potatoes	1.0E-02^b^	5.0^c^	0.019	0.036
Leafy vegetables	3.0E-03_soil_^b^	3.0^d^	0.106	0.203
Other vegetables/	8.0E-04 × [Pb]_soil_^b^	3.3^d, e^	0.044	0.084

^a^Dudka et al. (1996); ^b^Attanayake et al. (2014); ^c^UK_GOV (2018); ^d^STATIS (2011); ^e^Zucchini as an example

Together with the wash-out (Eq. 5), Eq. 6 becomes

7 $$\frac{d{[Pb]}_{\mathrm{soil}}}{dt}=IN-6\times {10}^{-3}{a}^{-1}\times {\left[Pb\right]}_{\mathrm{soil}}.$$

Note: the input (IN) has to be expressed as mg/(kg × a)*.*For [Pb]_*t*=0_ ≠ 0, the exact solution of Eq. (7) is

8 $${[Pb]}_{\mathrm{s}\mathrm{o}\mathrm{i}\mathrm{l}, t}=\frac{IN}{0.006}\times \left\{1-\mathrm{exp}\left(-0.006\times t\right)\right\}+\left\{{\left[\mathrm{P}\mathrm{b}\right]}_{\mathrm{s}\mathrm{o}\mathrm{i}\mathrm{l}, t=0}\times \mathrm{exp}\left(-0.006\times t\right)\right\},$$

with [Pb]_t=0_ being the Pb concentration in soil at the beginning of the observation period. This initial Pb concentration was set to 0.1 mg/kg for scenarios A1, B1, C1 and D1 (0.1 mg/kg as control level for crop-fields) and 70 mg/kg for scenarios A2, B2, C2 and C2 (70 mg/kg as precautionary level in silt soil).

### Oral exposure via crops

For the exposure via plant food, the EU food basket for vegetarians was taken as the basis for the calculation (EFSA 2010), which includes consumption of eggs and dairy products (Table 4). Data for modeling the exposure pathway soil–cow–milk or soil–hen–egg were not available; therefore, only the human exposure trend via direct consumption of crops was modeled.

**Table 4:** Plant food intake of adult vegetarians and children for different plant species

Plant species	Daily intake adult vegetarian [kg/(person × d)]^a^	Daily intake child [kg/(person × d)]^b^
Cereals	0.283	0.11
Potatoes	0.094	0.10
Leafy vegetables	0.318	0.19
Other vegetables	0.144	0.18

^a^EFSA (2010) (Table 21), daily intake for a vegetarian of 60 kg b.w.; ^b^recommended intake for a child of 20 kg b.w. (Kersting et al. 2017)

For children of 20 kg body weight (b.w.), the recommended daily food basket was taken as the basis for the calculations (Kersting et al. 2017). Although this includes meat, it was assumed that only direct crop intake adds to Pb exposure for children (Table 4). The daily uptake of Pb is estimated by multiplying these daily crop intakes with the relevant plant load of Pb; these plant loads are dependent on the Pb content on soil and the uptake factors (UF, Table 3) and are calculated with Eqs. (1)–(4). The daily oral exposure via plant products was calculated as

9 $${\text{Oral}}\;{\text{exposure}}\;{\text{by}}\;{\text{plant}}\;{\text{food}} = \left[ {{\text{Pb}}} \right]_{{{\text{soil}}}} \times \sum_{i} \left\{ {\left( {{\text{daily}}\;{\text{intake}}} \right)_{i} \times {\text{UF}}_{i} } \right\},$$

and the soil concentration of Pb was calculated according to Eq. (8).

### Risk evaluation

For risk evaluation, the daily uptake was compared to the reference values for different toxicological endpoints. The margin of safety (MOS) was defined for toxicological endpoints having a threshold, whereas the margin of exposure (MOE) was allocated to carcinogenic effects driven by genotoxicity:

$$\text{MOS or MOE}=\frac{\text{BMDL for toxicological endpoint}[\frac{\mathrm{m}\mathrm{g}}{\mathrm{k}\mathrm{g } \mathrm{b}.\mathrm{w}. \times \mathrm{d}}]}{\text{daily exposure}[\frac{\mathrm{m}\mathrm{g}}{\mathrm{k}\mathrm{g} \mathrm{b}.\mathrm{w}. \times \mathrm{d}}]}$$

The toxicity of Pb was summarized by reports of the US ATSDR (ATSDR 2007, 2019) and the EFSA (EFSA 2010). In terms of the respective BMDL, the most critical endpoints are neurodevelopmental toxicity in children and renal toxicity for adults. Kidney cancer may be identified as another critical endpoint, if Pb is to be regarded as a non-threshold carcinogen (see below).

For developmental neurotoxicity (dev.neu.), the dose–response analysis of the available epidemiological data resulted in a combined linear dose–response for oral exposure:

$${\text{BMDL}}_{{0{1},{\text{dev}}.{\text{neu}}.}} = {12}\;\mu {\text{g}}\;{\text{Pb}}/{\text{L}}\;{\text{blood}} = 0.{5}\;\mu {\text{g}}\;{\text{Pb}}/\left( {{\text{kg}}\;{\text{b}}.{\text{w}}. \times {\text{d}}} \right)$$.

(EFSA 2010; Budtz-Jorgensen et al. 2013).

Based on epidemiological data, the benchmark dose for chronic nephrotoxicity (neph.tox.) is,

$${\text{BMDL}}_{{0{1},\;{\text{neph}}.{\text{tox}}.}} = {15}\;\mu {\text{g}}\;{\text{Pb}}/{\text{L}}\;{\text{blood}} = 0.{63}\;\mu {\text{g}}\;{\text{Pb}}/\left( {{\text{kg}}\;{\text{b}}.\;{\text{w}}. \times {\text{d}}} \right)$$,

(EFSA 2010).

As Pb can increase kidney tumor incidences in perinatal exposed mice without overt chronic nephropathy (Waalkes et al. 1995), the lower 95% confidence interval for 10% excess risk for kidney tumors is,

$${\text{BMDL}}_{{{1}0\;({\text{mouse}},\;{\text{kidney}}\;{\text{cancer}})}} = {3}0.{5}\;{\text{mg}}\;{\text{Pb}}/{\text{kg}}\;{\text{b}}.{\text{w}}./{\text{d}}$$;

with an oral scaling factor of 7, for human beings of 60 kg b.w., the result is,

$${\text{BMDL}}_{{{1}0,\;{\text{human}}\;{\text{beings}}}} = {519}\;{\text{mg}}/\left( {{\text{person}} \times {\text{d}}} \right)$$

(see Supplemental Information).

For developmental neurotoxicity, as well as for nephrotoxicity, an MOS of 1 is deemed tolerable, as both endpoints are based on a broad epidemiological database. For kidney cancer, a minimum MOE of 10,000 is required. On this basis, a reverse-calculation may be run to estimate after how many years the Pb load in soil is so high that the tolerable daily intake (TDI) is met.

The corresponding Pb content in soil and the time interval to arrive that level are called [Pb]_soil, crit_. and t_crit_.

10 $${\text{TDI}}\left[ {\mu {\text{g}}/\left( {{\text{person}} \times {\text{d}}} \right)} \right] = \sum_{i} \left( {m_{i} \times {\text{UF}}_{i} } \right) \times \left[ {{\text{Pb}}} \right]_{{{\text{soil}},\;{\text{crit}}.}} \leftrightarrow \left[ {{\text{Pb}}} \right]_{{{\text{soil}},\;{\text{crit}}.}} = {\text{TDI}}/\left\{ {\sum_{i} \left( {m_{i} \times {\text{UF}}_{i} } \right)} \right\},$$

with [Pb]_soil, crit._ being the critical Pb concentration in soil, m the mass of plant ingested per day and UF its uptake factor, summed up for each plant food species *i*. These calculations assume a constant, non-changing Pb input on soil over prolonged periods of time, and that plant food is the sole source of Pb exposure.

If in Eq. 8, t is very high, equilibrium will be established, and

11 $$\left[ {{\text{Pb}}} \right]_{{{\text{soil}},t \to \infty }} = {\text{IN}}/{\text{k}} = {\text{IN}}/0.00{6}.$$

If ${[Pb]}_{\mathrm{s}\mathrm{o}\mathrm{i}\mathrm{l}, t\to \infty }>{[Pb]}_{\mathrm{s}\mathrm{o}\mathrm{i}\mathrm{l}, \mathrm{c}\mathrm{r}\mathrm{i}\mathrm{t}}$, the scenario calls for reduction of Pb input into soil. Rearrangement of Eq. 8 allows to calculate the time period after which the critical soil concentration would be arrived at

12 $${t}_{\mathrm{c}\mathrm{r}\mathrm{i}\mathrm{t}}=-\frac{ 1}{k}\times \left\{\mathrm{L}\mathrm{N}\left({\left[\mathrm{P}\mathrm{b}\right]}_{\mathrm{s}\mathrm{o}\mathrm{i}\mathrm{l}, \mathrm{c}\mathrm{r}\mathrm{i}\mathrm{t}}-\frac{\mathrm{I}\mathrm{N}}{k}\right)-\mathrm{L}\mathrm{N}\left({\left[\mathrm{P}\mathrm{b}\right]}_{\mathrm{s}\mathrm{o}\mathrm{i}\mathrm{l}, t=0}-\frac{\mathrm{I}\mathrm{N}}{k}\right)\right\}.$$

## Results

### Pb input into soil

**Fig. 2:**
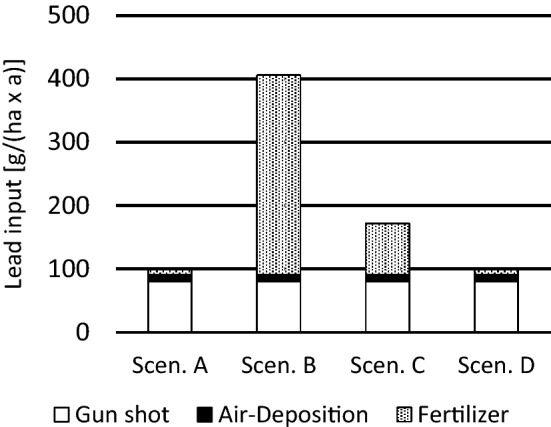
Annual Pb input on fields by gun shot, air deposition and fertilizer, g/(ha × a) for four different farming scenarios (Knappe et al. 2008): Scenario **A** use of cattle manure and mineral fertilizer; Scenario **B** use of compost and mineral fertilizer; Scenario **C** use of sewage sludge and mineral fertilizer; Scenario **D** exclusive use of mineral fertilizer

The annual Pb input into agricultural soil was calculated using the medians and best estimates for deposition by (1) gunshot (80 g/(ha × a), (2) background air deposition (11 g/(ha × a) and (3) fertilizer input. The contribution of fertilizer was based on a previous study that estimated Pb input into soil for four typical scenarios of conventional farming (Knappe et al. 2008). These four conditions, further named scenarios A–D, were calculated to cause a Pb input by fertilizer of 7.5, 313.0, 69.5 or 6.3 g/(ha × a), and for all three sources a total Pb input of 98.5, 405.6, 171.7 and 98.8 g/(ha × a), respectively (Fig. 2). The highest Pb input on soil was obtained for scenario B, the lowest (and very similar) inputs for scenarios A and D, while scenario C was intermediate.

### Time-dependent simulation of Pb concentrations in farmland

**Fig. 3:**
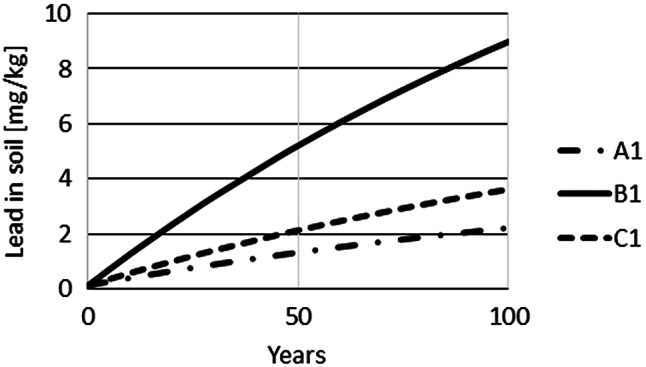
Simulation of Pb concentrations in soil for 100 years for Pb input scenarios A1, B1 and C1

**Fig. 4:**
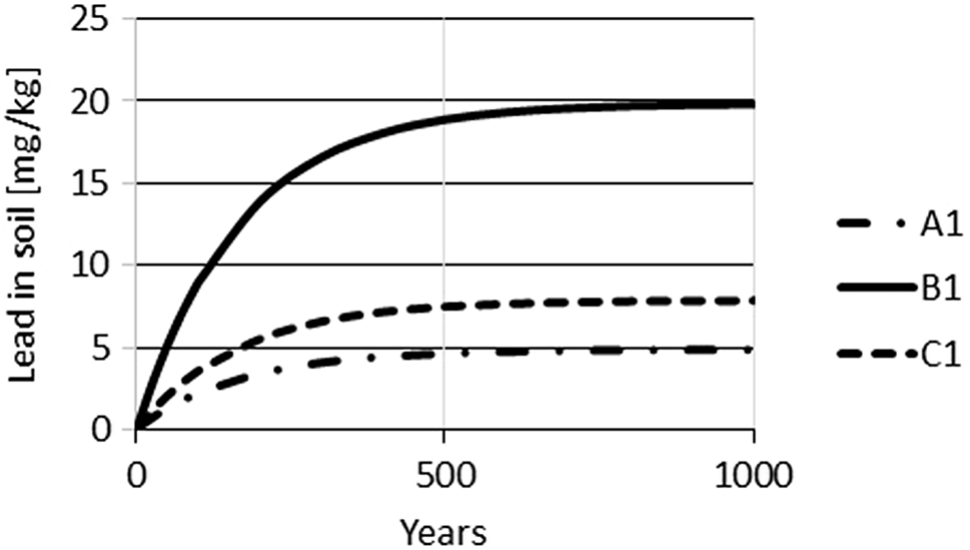
Long-term simulation of Pb concentrations under the same input scenarios shown in Fig. 3

**Fig. 5:**
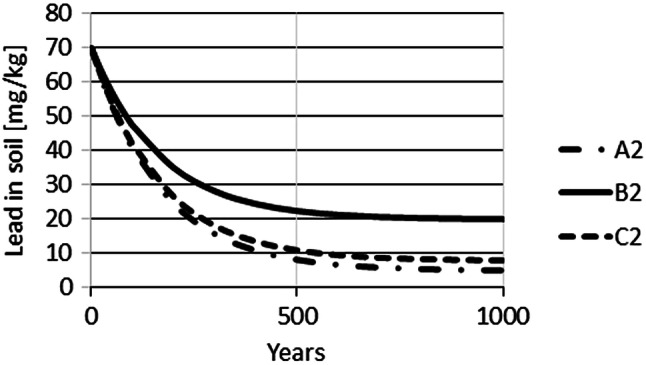
Time-dependent Pb concentration in soil for scenarios A2, B2 and C2 for constant Pb input

The time-dependent Pb concentrations in farmland were simulated using Eq. (8). For these simulations, a constant input of Pb on soil was assumed for very long periods. As the Pb input for farming scenarios A and D is very similar, the following calculations were only performed for farming scenarios A–C. The initial soil concentration of Pb was set to (1) 0.1 mg/kg, the control level for crop fields; and (2) 70 mg/kg, the precautionary level for silt-soil. Therefore, a total of 6 scenarios were simulated, A1–C1 with initial soil concentrations of 0.1 mg/kg and A2–C2 with initial soil concentrations of 70 mg/kg. Simulations of time-dependence show that approximately 100 years are required until Pb concentrations increase from an initial concentration of 0.1 mg/kg (mg/kg) to 2 (A1), 4 (C1) or 9 (B1) mg/kg (Fig. 3). The simulation also demonstrates that changes within the first 10 years are relatively small, not exceeding an increase of 2 mg/kg for all three scenarios. Equilibrium levels for Pb of approximately 5, 20 and 8.5 mg/kg in soil were predicted to be reached for scenarios A1, B1 and C1, respectively, only after about 600 years (Fig. 4). If the initial concentration is set to 70 mg/kg, planting and harvest result in a reduction of Pb in soil over time until the same equilibrium concentrations would be reached as for the staring situation with 0.1 mg/kg. However, approximately 600 years would be required until these equilibrium levels are achieved (Fig. 5).

### Critical soil levels

The oral exposure of humans due to consumption of crops was simulated for the different scenarios. As 70 mg/kg Pb content in soil is above the control level of the German Soil Protection Ordinance, we focused on scenarios A1, B1, C1 and D1, in which the initial soil concentration is 0.1 mg/kg.

Using Eq. 10 and data from Tables 3 (UF), 4 (m) and 5, the critical soil concentration, [Pb]_soil_,_crit_,_._ was calculated (Table 6).

**Table 5:** TDI values [µg/(person × d)] as defined in the text

Toxicological Endpoint	TDI Child^a^ [µg/(person × d)]	TDI Adult^b^ [µg/(person × d)]
Developmental Neurotoxicity	10	–
Renal failure	–	38
Kidney cancer	–	52

^a^20 kg b.w.; ^b^60 kg b.w

**Table 6:** Critical soil concentrations according to Eq. 10

Toxicological Endpoint	TDI [µg/(person × d)]	[Pb]_soil, crit._[mg/kg]
Developmental Neurotoxicity	10^a^	5
Renal failure	38^b^	18
Kidney cancer	52^b^	25

The Pb concentrations in soil are given (Pb_soil_, mg/kg) that were simulated to correspond to the specific tolerable daily intake (TDI) for three toxicological endpoints ^a^20 kg b.w.; ^b^60 kg b.w

**Fig. 6:**
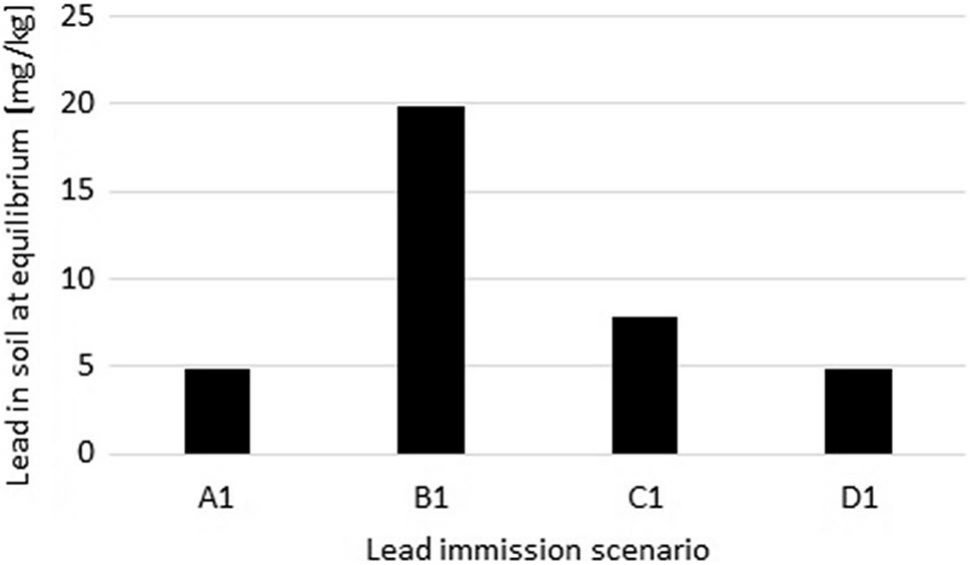
Equilibrium soil concentration of Pb according to four farming scenarios

Equilibrium concentrations of Pb according to the farming scenario were calculated using Eq. 11 (Fig. 6). For scenario B1, the equilibrium concentration exceeds the critical soil concentration of Pb for both renal failure and developmental neurotoxicity, whereas scenario C1 exceeds the critical soil concentrations of Pb for developmental neurotoxicity only.

**Fig. 7:**
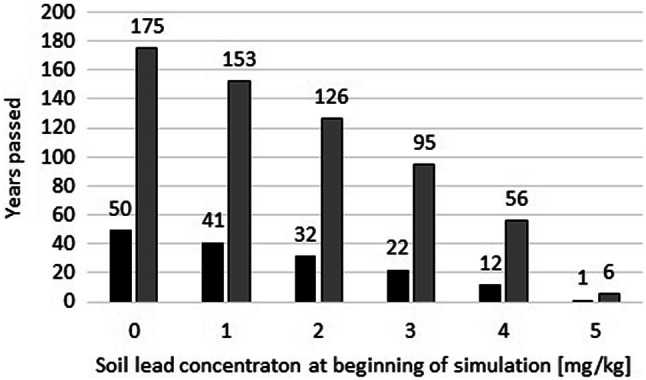
Time periods in years until critical soil concentration of Pb would be exceeded with respect to developmental neurotoxicity. The simulations are based on farming scenario B1 (black bars) and C1 (grey bars). The initial Pb concentration in soil are given on the x-axis

It is important to predict the number years of a specific Pb input required for critical soil concentrations for developmental neurotoxicity to be exceeded. Therefore, simulations for scenario B1 and C1 were performed with different initial Pb concentrations in soil as a starting situation (Fig. 7). Assuming a concentration of 0.1 mg/kg Pb in soil, approximately 50 years of Pb input according to scenario B1 would be required to exceed the critical soil concentration; for scenario C1, this period increases to approximately 175 years. With increasing initial Pb concentrations, the periods gradually decrease (Fig. 7). For an initial Pb concentration of 5 mg/kg, the respective periods are only 1 and 6 years for scenarios B1 and C1, respectively. With the lowest critical level of Pb in soil of 5 mg/kg, the annual input should not exceed about 10 mg/(m^2^ × a) = 100 g/(ha × a) in order not to exceed critical soil concentrations.

## Discussion

In recent years, human exposure to Pb has been decreased by several measures (Wietlisbach et al. 1995; Pirkle et al. 1994). Nevertheless, there is still persistent immission of Pb onto agricultural soils; the major sources are from the use of fertilizer, Pb ammunition from hunting activities, and Pb from air deposition. Little is known whether current immission is high enough to cause an increase of Pb concentrations in agricultural soil over prolonged periods. If so, the questions raised are regarding the level of the resulting equilibrium concentration, the time span until equilibrium would be established, and whether critical concentrations would be reached for consumers of agricultural products. To answer these questions, we performed simulations of Pb concentrations in soil, using different previously reported input scenarios. A main result of these simulations is that current input scenarios would require very long periods of approximately 600 years until new equilibrium concentrations would be established, where the Pb input equals Pb removal by the harvest of plants and by washout. We calculated that a concentration of ~ 5 mg/kg Pb in agricultural soil leads to an intake of approximately 10 µg Pb per person per day by consumption of representative amounts of agricultural products obtained from this soil; 10 µg per person per day is considered as the tolerable daily intake (TDI) based on data on developmental neurotoxicity. Therefore, the concentration of 5 mg/kg Pb was considered a critical concentration in soil that should not be exceeded. Starting with a soil concentration of 0.1 mg/kg, the control level for crop fields, our simulation predicts periods between 50 and 175 years until the critical concentration of 5 mg/kg Pb in soil would be reached, depending on the immission scenario.

In our model, air deposition, application of fertilizer and hunting with gunshot were included as sources for Pb input in agricultural soil. For fertilizers, compost is the major contributor of Pb (Knappe et al. 2008), and for scenario B, the relevance of Pb sources is fertilizer > hunting > air deposition. For scenarios A, C and D, hunting with gunshot is the major contributor for Pb input in soil, while fertilizer contributes most in scenario B. As our calculations demonstrate, it may take several hundreds of years before Pb in soil has achieved an equilibrium starting from boundary values of 0 or 70 mg/kg. Although several measures were taken to reduce the Pb input into soil over the last decades, it is likely that equilibria have not yet been reached. It is unlikely that Pb concentrations in agricultural soil will decrease over the next decades; contrary to this, soils with currently low Pb concentrations may exceed critical levels in the future. It should be considered that all predictions of the present study were made under the assumption that the Pb input on soil will not change in the future. In Europe, Pb in soil ranges from < 10 to > 70 mg/kg, with a median of 23 mg/kg (EFSA 2010). According to our model, the lowest critical soil level is 5 mg/kg; the maximum permissible input on soil should, therefore, not exceed 10 mg/(m^2^ × a), corresponding to 100 g/(ha × a); these conditions are fulfilled in scenarios A1 and D1. A maximum input of 100 g/(ha × a) should also be protective with respect to renal failure and kidney cancer, endpoints, for which data show the effects only with higher doses than for development neurotoxicity. However, the conclusion is based on the assumption that plant food is the sole source for Pb exposure. The German Federal Soil Protection and Contaminated Sites Ordinance (BMJV 2017) currently names a maximum permissible input of 400 g Pb per ha and year, which is only slightly exceeded by scenario B1.

A further question addressed by the simulations is the time period required until the Pb concentration in contaminated soil is reduced to lower equilibrium levels. For this purpose, scenarios A2, B2 and C2 were applied, starting at relatively high levels of Pb in soil. Uptake of Pb by plants may reduce its concentrations in soil, a mechanism known as phytoremediation (Kushwaha et al. 2018). The simulations demonstrated that several centuries would be required until lower equilibrium concentrations of Pb in soil would be established.

Whether or not Pb is a non-threshold genotoxic carcinogen is debatable. The German MAK Commission classified inorganic Pb compounds as carcinogen MAK-category 2 (equivalent to GHS category 1B); (DFG 2009). In their evaluation, EFSA considered Pb as a weak, probably indirect mutagen, increasing kidney tumor incidents in rodents at comparatively high dosages (EFSA 2010). The International Agency for Research on Cancer concluded that there is only limited evidence for direct DNA interaction, whereas inhibition of DNA repair and oxidative stress are involved in Pb genotoxicity (IARC 2006). In any case, we estimated the BMDL_10_ for kidney cancer based on the data of Waalkes et al. (1995) and used this as a point of departure for the MOE calculation.

The models used for our simulations have several limitations and contain assumption and simplifications. First, we assumed a linear relationship between Pb uptake by plants and the Pb content in soil. This assumption is debatable. According to Knappe et al. (2008), Pb content in soil results in Pb in plants but the quantitative relationship may not necessarily be linear. For example, in curly kale, 20 mg/kg Pb in the soil resulted in plant contents between 1 and 10 mg/kg, and ~ 60 mg/kg Pb in soil resulted in measured plant contents of 1–35 mg/kg. Using our model, the calculated content for leafy vegetables was 0.03 and 0.18 mg/kg for soil contents of 10 and 60 mg/kg, respectively, which is lower compared to Knappe et al. (2008). Wang et al. (2006) investigated Pb uptake in vegetables and derived a sublinear relationship, for example for leafy vegetables, ${[\mathrm{P}\mathrm{b}]}_{\mathrm{p}\mathrm{l}\mathrm{a}\mathrm{n}\mathrm{t}}=0.03\times {[Pb]}_{\mathrm{s}\mathrm{o}\mathrm{i}\mathrm{l}}^{0.33}$. This relation will result in a saturated plant-uptake for increasing Pb concentrations in soil. Using this formula for leafy vegetables would result in a 14-fold higher Pb content in the plant for 0.1 mg/kg Pb in soil, and 2.5-fold lower Pb content in the plant for 20 mg/kg Pb in soil, compared to our model. Since Wang et al. (2006) have not published data for cereals and root crops, and because Attanayake et al. (2014) and Dudka et al. (1996) worked with partly highly contaminated soils, we decided to apply a linear uptake model in the present study. Taken together, our model tends to underestimate oral exposure to Pb via plant food than to overestimate it. Second, the input of Pb by gunshot was assumed to be homogenous in the present model, which in reality is not the case. The bullets present dispersed Pb centers in the soil, where they disintegrate (Rooney et al. 2007); the Pb input on soil by gunshot is a rough estimate, based on market volumes and the potential hunting area in the EU. More robust immission data are desirable for a more robust estimate of the contribution of gunshot to soil Pb burden. Third, we assumed that the top 20 cm soil layer is relevant for mixing and exchange of Pb. Nevertheless, although we have introduced these simplifications, Pb content in crops and Pb in soil modeled with scenarios A, B, C and D do match measured values reported in other publications (EFSA 2010, 2012; Toth et al. 2016). Concerning oral exposure to Pb, plant products cover up to 40% and beverages up to 23% of the burden for adults; the median oral exposure in the EU is estimated to be approximately 0.9, 1.3, 1.0 and 0.5 µg/kg b.w./day for infants, toddlers, other children and adults, respectively; the 95-percentile was estimated to be approximately 1.8, 2.2, 1.7 and 0.8 µg/kg b.w./day for infants, toddlers, other children and adults, respectively (EFSA 2012). Forth, we assumed that cooking does not reduce the Pb load in plant food, which is a reasonable assumption considering that food preparation does not significantly reduce the Pb content in fish (Diaconescu et al. 2013). Only in the case of the preparation of acidified food it is possible that some Pb is extracted from the plant material. Finally, it should be considered that besides the simulated sources (air deposition, fertilizer and gunshot), humans may also be exposed to Pb via drinking water. The limit value for Pb in drinking water is 10 µg/L (WHO 2017; TrinkwasserVO 2018). With a daily consumption of 1–2 L per person and day, this potential additional source of Pb exposure can reduce the margin of safety calculated here. Further, Pb in soil may contribute to the Pb burden in ground water. However, the drift velocity of Pb anions in soil is slow; therefore, Pb dispersed onto soil may cause problems not earlier than after several decades (see Supplemental Information).

Measures to reduce future oral Pb exposure via plant food should address the Pb content in compost. This problem was also investigated by other authors, including Dai et al. (2006), Singh et al. (2010), Lopes et al. (2011) and Eid et al. (2017). Methods for heavy metal reduction in organic material and sludge have been published (e.g., Vogel et al. 2013). Such processes are sophisticated and require technical and financial efforts. Over the last two decades, the median level of Pb in compost decreased from 52 mg/kg (d.w.) in 1999, to 31, 28.3 and 27.0 mg/kg (d.w.) in 2012, 2015 and 2018, respectively (Hermann et al. 2017; Bundesgütegemeinschaft-Kompost 2019).

Next to compost, hunting with gunshot is the most important contribution of Pb input into agricultural soil. Substitution of Pb in hunting ammunition by less problematic materials would not only reduce oral Pb exposure via plant food, but also reduce exposure caused by the consumption of game meat (Pain et al. 2010; Mueller-Graf et al. 2017).

Due to the severe toxic effects caused by Pb, it is recommended to closely supervise future developments in Pb levels in the environment and the dietary Pb exposure of consumers. Comparatively simple measures to reduce the long-term exposure to Pb are possible and should not be postponed.

## Electronic supplementary material

Below is the link to the electronic supplementary material.

Supplementary file1 (DOCX 25 kb)
