# Supplementary material for: Long-term simulation of lead concentrations in agricultural soils in relation to human adverse health effects
Source: Arch Toxicol. 2020 May 5;94(7):2319–29. doi: 10.1007/s00204-020-02762-x (PMC7367917; doi:10.1007/s00204-020-02762-x)
Supplement: Supplementary file 1 — Supplementary file1 (DOCX 25 kb) [file 204_2020_2762_MOESM1_ESM.docx]

# SUPPLEMENTAL INFORMATION

# Long-term simulation of lead concentrations in agricultural soils in relation to human adverse health effects

## Benchmark Dose Software v3.0 and PROAST v67.0 Evaluation of Pb-induced renal tumors in in mice

Waalkes et al. (1995) published data on renal tumors in mice which were perinatally exposed to lead acetate (Pb(OAc)_2_) via drinking water (Table A-1). For the benchmark dose analysis, two programs are publicly available: the Benchmark Dose Software (BMDS) of the United States Environmental Protection Agency (US EPA 2019) and the program PROAST (RIVM 2012, 2019).

Evaluation of these data with the Benchmark Dose Software v3.0 (US EPA 2019) delivered a BMDL_10_ of 519 mg/L (ppm) Pb(OAc)_2_ for the multistage degree 2 model, which is the recommended output for the data analyzed by the program. A value of 519 mg/L Pb(OAc)_2_ is equivalent to 331 mg/L Pb^2+^. The mouse has a daily consumption of drinking water of about 183 mL water per kg b. w. (ECHA 2012); therefore, the BMDL_10_ for the mouse is:

BMDL_10, mouse_ = 60.6 mg/(kg b. w. × d).

For oral exposure in terms of mg/(kg b. w. × d), the scaling factor from mouse to man is 7. For a person with a body weight of 60 kg, the benchmark dose limit for human beings is:

BMDL_10, human_ = 519 mg/(person × d).

For information, the analysis output of the PROAST program is provided in Table A-2. Concerning the lowest BMDL, there is very little difference between the programs; therefore, we have not discussed which program is preferred.

## Drift of Pb in soil

The drift speed of Pb in soil is between 0.004 and 0.016 m per year (Chrastny et al. 2014). A part of the Pb dispersed on soil may eventually end up in groundwater. If the relevant groundwater level is used as drinking water resource, Pb spread on soil may contribute to oral Pb exposure via drinking water. A net water flow from the soil surface to groundwater levels will finally cause a Pb burden to groundwater. In Germany, the 90-percentile background level of Pb in groundwater is 1.05 µg/L (LAWA 2015). The median annual groundwater renewal rate in Germany is 100 L/m² (Neumann and Wycisk 2002). Pb ions in the surface water will be in equilibrium with its poorly soluble salts, with adsorbents like clay minerals, with iron and manganese oxides and with chelating ligands in soil organic material (Kushwaha et al. 2018). Nevertheless, Pb will drift towards the groundwater layer at a rate of 0.004 to 0.016 m/a (Chrastny et al. 2014). This “Pb-soil-chromatography” peak will eventually reach groundwater layers. Table A-4 lists the retention time depending on the distance and the drift velocity. A long time may pass before the Pb-peak in infiltrating water arrives at critical ground water layers. Areas with a previously comparatively high input of Pb into soil would call for a long-term monitoring of the groundwater even if more recent Pb deposition is deemed to be of low relevance.

## References

Chrastny V, Vanek A, Cadkova E, Ruzickova A, Galuskova I, Faturikova D, Komarek M (2014) Lead migration in a smelter impacted deciduous and coniferic soil horizons based on a long-term in-situ implantation and laboratory column experiments. Applied Geochemistry 48:168-175. http://dx.doi.org/10.1016/j.agrochem.2014.06.027

ECHA (2012) Guidance on information requirements and chemical safety assessment. Chapter R.8: Characterisation of dose [concentration]-response for human health. European Chemicals Agency. [https://echa.europa.eu/documents/10162/13632/information_requirements_r8_en.pdf/e153243a-03f0-44c5-8808-88af66223258](about:blank). Accessed 06 May 2019.

Kushwaha A, Hans N, Kumar S, Rani (2018). A critical review on speciation, mobilization and toxicity of lead in soil-microbe-plant system and bioremediation strategies. Ecotoxicology and Environmental Safety 147:1035-1045. <https://doi.org/10.1016/j.ecoenv.2017.09.049>

LAWA (2015) BLA-GEO Hydrogeochemische Hintergrundwerte im Grundwasser und ihre Bedeutung für die Wasserwirtschaft. Bund-Länder-Ausschuss Bodenforschung und Länder Arbeitsgemeinschaft Wasser. 2015. [https://www.bgr.bund.de/DE/Themen/Wasser/Projekte/abgeschlossen/Beratung/Hintergrundwerte/sgd&lawa2015.pdf?__blob=publicationFile&v=4](about:blank). Accessed 03 May 2018.

Neumann J, Wycisk P (2002) Mittlere jährliche Grundwasserneubildung. In: Bundesanstalt für Geowissenschaften und Rohstoffe (ed) Nationalatlas Bundesrepublik Deutschland - Relief, Boden und Wasser, Leipzig, 2002; pp. 144-145. [archiv.nationalatlas.de/wp-content/art_pdf/Band2_144-145_archiv.pdf](about:blank). Accessed 05 May 2018.

RIVM (2012, 2019) Rijksinstituut voor Volksgezondheit en Milieu (National Institut for Public Health and the Environment, The Netherlands). PROAST version 67.0. [https://www.rivm.nl/en/proast](about:blank). Accessed 12 December 2019.

US EPA (2019) Benchmark Dose Software. Version 3.0. United States Environmental Protection Agency. [https://www.epa.gov/bmds.](about:blank) Accessed 19 January 2019.

Waalkes MP, Diwan BA, Ward JM, Devor DE, Goyer RA (1995) Renal tubular tumors and atypical hyperplasias in B6C3F1 mice exposed to lead acetate during gestation and lactation occur with minimal chronic nephropathy. Cancer Research 55:5265-5271. doi: Published November 1995

**Tab. A-1:** Renal tumors in mice perinatally exposed to lead acetate {Pb(OOC-CH_3_)_2_} in drinking water (Waalkes et al. 1995)

| Dose [mg/L] in drinking water | No. of exposed animals | Renal tubular adenoma or carcinoma |
| --- | --- | --- |
| 0 | 23 | 0 |
| 500 | 25 | 1 |
| 750 | 25 | 1 |
| 1000 | 25 | 5 |

**Tab. A-2:** Benchmark Dose software v3.0, analysis of kidney tumor data in mice, perinatally exposed to lead acetate {Pb(OOC-CH_3_)_2_} in drinking water (mg/L) (Waalkes et al. 1995); 10 % extra risk, 95 % confidence interval..

| Model | BMD | BMDL | P Value | AIC | BMDS Recommendation |
| --- | --- | --- | --- | --- | --- |
| Gamma | 908.87 | 519.14 | 0.3282 | 49.127 | Viable - Alternate |
| Log-Logistic | 811.60 | 541.89 | 0.6295 | 46.798 | Viable - Alternate |
| Multistage Degree 3 | 813.36 | 549.54 | 0.6630 | 46.731 | Viable - Alternate |
| Multistage Degree 2 | *798.23* | *518.62* | *0.7591* | *45.1305* | *Viable - Recommended* |
| Multistage Degree 1 | 798.65 | 454.40 | 0.4851 | 46.3276 | Viable - Alternate |
| Weibull | 836.41 | 680.83 | 1.59E-05 | 67.3089 | Questionable |
| Dichotomous Hill | 811.60 | 6.9E-08 | 0.3360 | 48.7979 | Questionable |
| Logistic | 846.55 | 698.12 | 0.6897 | 46.5866 | Viable - Alternate |
| Log-Probit | 798.45 | 3.06E-08 | 0.5919 | 46.9622 | Questionable |
| Probit | 829.68 | 670.31 | 0.6691 | 46.6624 | Viable - Alternate |

**Tab. A-3:** PROAST 67.0 analysis of kidney tumor data in mice, perinatally exposed to lead acetate {Pb(OOC-CH_3_)_2_} in drinking water (mg/L) (Waalkes et al. 1995); 10 % extra risk; constraint on steepness parameter: 0.01; critical AIC value: 2

| Model | BMDL^a)^ | BMDU^b)^ | BMD^c)^ | AIC^d)^ | accepted |
| --- | --- | --- | --- | --- | --- |
| Null | NA^e)^ | NA | NA | 52.44 | No |
| Full | NA | NA | NA | 49.82 | No |
| Two stage | 602 | 1320 | 798 | 49.14 | Yes |
| Log logistic | 539 | 1280 | 812 | 48.80 | Yes |
| Weibull | 542 | 1260 | 815 | 48.76 | Yes |
| Log prob. | 520 | 1390 | 798 | 48.76 | Yes |
| Gamma | 530 | 1310 | 806 | 48.86 | Yes |
| Logistic | NA | NA | NA | 54.34 | No |
| Probit | 655 | 1110 | 863 | 47.24 | Yes |
| Expo m3 | 558 | 1210 | 836 | 48.66 | Yes |
| Hill m3 | 550 | 1240 | 832 | 48.72 | Yes |

^a)^: lower 95 % confidence limit; ^b)^: upper 95 % confidence limit; ^c)^: Benchmark dose; ^d)^: Akaike’s Information Criteria; ^e)^: not applicable;

**Tab. A-4:** Correlation of the retention time of Pb^2+^ (years) in soil with the relevant depth of groundwater layer and drift velocity (Chrastny et al. 2014)

| Relevant soil depth | Drift velocity | |
| --- | --- | --- |
|  | 0.004 m / a | 0.016 m / a |
| 2 m | 500 a | 125 a |
| 10 m | 2500 a | 625 a |
| 20 m | 5000 a | 1250 a |
| 40 m | 10000 a | 2500 a |
